# Soil samples from sporotrichosis transmission belt area: Searching for fungal species and their antagonistic activity against *Sporothrix brasiliensis*

**DOI:** 10.3389/fcimb.2022.1033969

**Published:** 2022-12-01

**Authors:** Gisela Lara da Costa, Isabella Escórcio Ferreira, Danielly Corrêa-Moreira, Anna Marinho, Adilson Benedito de Almeida, Sandro Antônio Pereira, Cintia Moraes Borba, Manoel Marques Evangelista Oliveira

**Affiliations:** ^1^ Laboratory of Taxonomy, Biochemistry and Bioprospecting of Fungi, Oswaldo Cruz Institute, FIOCRUZ, Rio de Janeiro, Brazil; ^2^ Postdoctoral in Clinical Research in Infectious Diseases, Evandro Chagas National Institute of Infectious Diseases, FIOCRUZ, Rio de Janeiro, Brazil; ^3^ Laboratory of Clinical Research in Dermatozoonoses in Domestic Animals, Evandro Chagas National Institute of Infectious Diseases, FIOCRUZ, Rio de Janeiro, Brazil

**Keywords:** environment, soil, fungal interaction, phenotypic characterization, *Sporothrix* spp., sporotrichosis, *Purpureocillium lilacinum*

## Abstract

Since 1998, the state of Rio de Janeiro, Brazil, has become a public health problem regarding sporotrichosis, a disease caused by *Sporothrix* spp. involving contact with infected cats. Efforts to isolate these species from environmental sources are not always successful. In our study, soil from residences situated in cities of Rio de Janeiro where cats with sporotrichosis live was collected and cultured an attempt to isolate *Sporothrix* spp. but it was not successful. However, other saprophytic fungal species were isolated from soil and identified and among them *Purpureocillium lilacinum* was the most frequent. From there, we decided to study the *in vitro* interaction of this species with *S. brasiliensis*, the principal agent that causes sporotrichosis in this state. The results showed that ten isolates of *P. lilacinum* inhibited the radial mycelial growth of *S. brasiliensis* with different percentage of inhibition. The interaction between them revealed the pattern described as overgrowth by antagonist. In conclusion, our data suggest that fungal species with very fast growth and capable of producing metabolites could hinder the growth of *Sporothrix* spp., it also opens the way for the identification of secondary metabolites with biological activity that could be tested against pathogenic fungi.

## Introduction

Sporotrichosis is a subacute or chronic infection caused by the thermodimorphic species of the genus *Sporothrix*. It is a cosmopolitan disease of tropical and subtropical regions and is the most frequent subcutaneous mycosis in Latin America (Orofino-Costa et al., 2017).

Since 1998 this mycosis has become a public health problem in the state of Rio de Janeiro, Brazil, due to the significant increase in human and feline cases (Schubach et al., 2008; Gremião et al., 2020). In general, in Brazil, the transmission of this mycosis is by traumatic inoculation of fungi with the handling of organic matter. However, in the great metropolitan area of Rio de Janeiro was observed a zoonotic transmission from infected cats to humans (Orofino-Costa et al., 2017).

An exploratory analysis of the socio-spatial distribution of sporotrichosis in Rio de Janeiro state, from 1997 to 2007, identified a transmission belt along the border between the city of Rio de Janeiro and the adjacent municipalities in the greater metropolitan area (Silva et al., 2012). Recently, the epidemiological bulletin on sporotrichosis in this same state showed cases of sporotrichosis in municipalities in all administrative regions of RJ (Brasil, 2021). Molecular analysis clearly demonstrated that *Sporothrix brasiliensis* is the primary cause of zoonotic disease in Rio de Janeiro state (Oliveira et al., 2011; Gremião et al., 2020).

According to Poester et al. (2018) the environment can have a function as a reservoir for *S. brasiliensis* in hyperendemic areas of feline sporotrichosis, which may transfer the fungus from the sick animal to the environment by contact of animal lesions with surrounding; by feces of sick felines; or by burying the bodies of sick felines. However, environmental studies with isolation of *Sporothrix* species are not performed frequently and when done the results are not always successful. The reasons why there are difficulties in isolating *Sporothrix* species from soil are still not clear, but it is known that saprophytic species can have antagonistic actions on the growth of each other (Boddy and Hiscox, 2016).

The aims of this study were to evaluate the presence of *Sporothrix* spp. in soil samples from areas considered to be the transmission belt of sporotrichosis in the Rio de Janeiro and analyze antagonistic activities of saprophytic fungi against *S. brasiliensis*.

## Materials and methods

### Soil samples

The samples were collected from soil of residences, in which cats with sporotrichosis live, located in Atlantic Forest areas, in the cities of Petrópolis and Vassouras, metropolitan and central-south regions of Rio de Janeiro state, respectively. These areas are situated in the state’s sporotrichosis transmission belt (Silva et al., 2012; Brasil, 2021). Approximately 50g of soil were collected 10 cm below the topsoil and stored in Falcon tubes to be transported to the laboratory.

### Isolation of fungi from soil samples

Fungi were isolated from soil using the serial dilution technique and inoculation in Petri dish containing potato dextrose agar medium (PDA – Difco, Becton-Dickinson and Company, New Jersey, EUA) with chloramphenicol (Adapted from Clark, 1965). Briefly, one gram of soil was weighed and placed in a 28 x 150 mm test tube. Sterile distilled water (10 mL) was added and the solution homogenized in vortex for 3 minutes. After, 1 mL of the soil solution was transferred to a test tube containing 9 mL of sterile distilled water, and so on to make serial dilutions (10^-1^ to 10^-5^). From the dilution 10^-3^ aliquots of 0.1 mL of solution was transferred to Petri dishes containing PDA (Difco) plus 0.05 g.mL^-1^ of chloramphenicol for inhibiting bacteria. They were placed in a BOD-type temperature chamber at 28°C ± 1°C, for seven days with daily monitoring.

The most representative colony forming units (CFU) were isolated and transferred to test tubes containing the same medium described above and kept in a BOD-type air-conditioned chamber with a controlled temperature of 28°C for future identification.

### Identification by phenotypic characteristics of the fungal isolates

The analysis of macroscopic characteristics was made using the PDA medium and incubation at 28°C. The colonies were measured with a Mitutoyo digital caliper (Mitutoyo America Corporation, Aurora, Illinois, USA) with a resolution of 0.01mm. Some characteristics that are relevant for identification were observed, such as: texture, coloration of conidia, coloration of mycelium and reverse, presence and characterization of exudate, soluble pigments (Barron, 1972; Barnett and Hunter, 1998).

Microcultures (Riddell, 1950) using PDA medium for 7 days, at 28°C, were done for micromorphological evaluation and species identification. The morphology was observed after staining with Amann lactophenol plus cotton blue solution and examined under a Nikon light microscope, E400 model (Nikon Instruments Inc., Melville, NY, United States).

### Screening by dual culture method

Antagonistic activity of the species isolated from soil was evaluated against *S. brasiliensis* type strain CBS120339 using dual culture assay (adapted from Liu et al., 2020). Using a platinum loop a constant quantity of fungi (the putative antagonist and *S. brasiliensis*) were separately point inoculated on PDA medium and placed 2 cm from the edge of the Petri dish at opposite ends. As a control, *S. brasiliensis* was placed in a similar manner on a fresh PDA plate. All pairings were carried out in triplicate and incubated at 28°C for seven days. Antagonistic activity was evaluated according to Rahman et al. (2009) by measuring the growth of both fungi in test and using the formula developed by Skidmore and Dickinson (1976): PIRG = R1 –R2/R1 x 100, where PIRG is percentage inhibition of radial growth; R1 is radius of the *S. brasiliensis* colony and R2 is radius of the putative antagonist colony. Interaction between species was monitored until the 28^th^ day of incubation at same temperature.

## Results

### Fungal species from soil samples

The total number of 143 CFU was obtained from the soil samples per municipality, 46 from Petrópolis (Pe-46) and 97 from Vassouras (Va-97). *Sporothrix* sp. was not isolated from soil samples.

**Figure 1:**
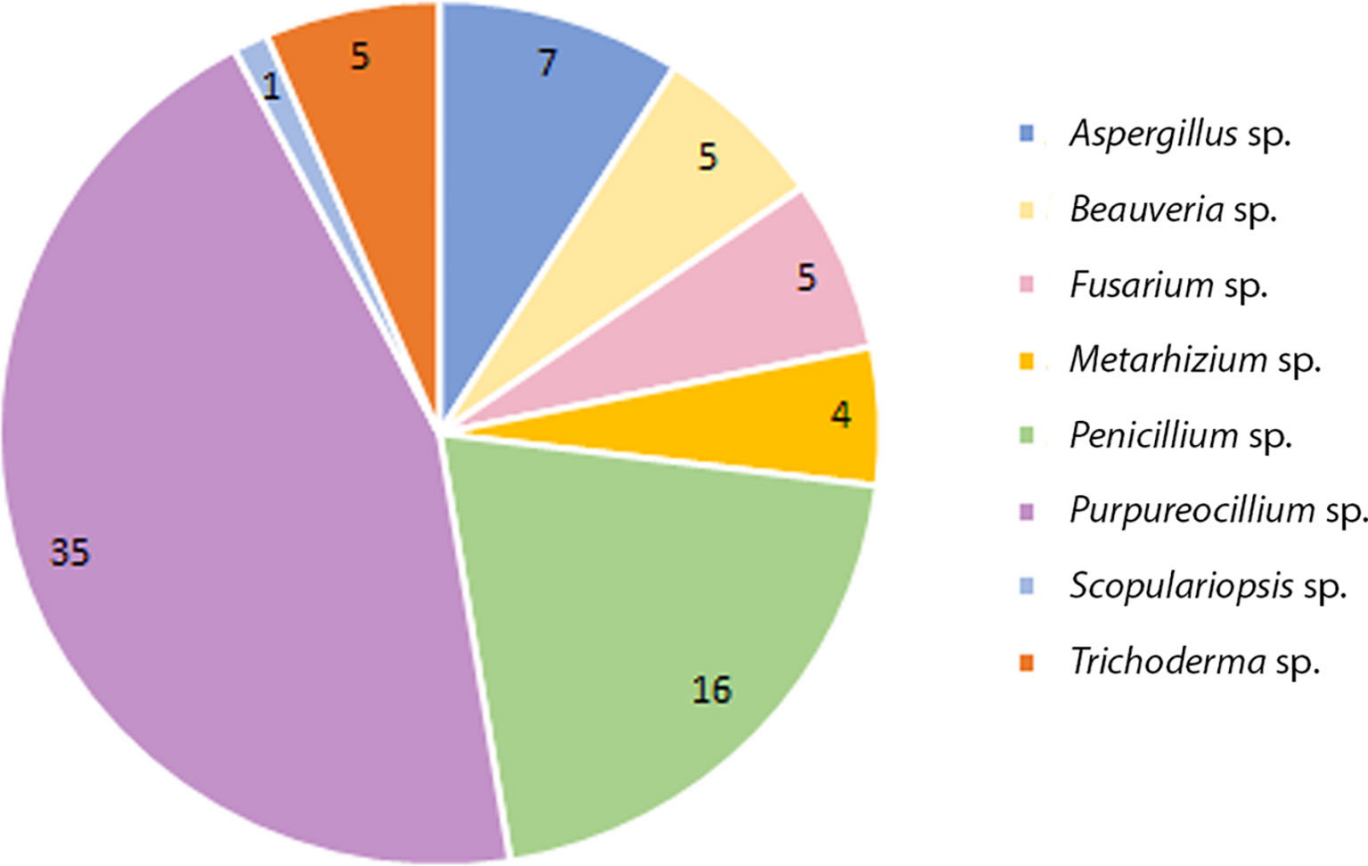
Total number of species collected from soil from domiciles with cases of feline sporotrichosis at Petrópolis and Vassouras, Rio de Janeiro state.

Of this total 137 colonies were filamentous fungi and 06 yeast-like colonies (not evaluated in the study). Eight genera were identified by classical taxonomy. *Purpureocillium* sp. presented the highest number of isolates 35 (Pe-6/Va-29), followed by *Penicillium* sp. with 16 (Pe-7/Va-9), *Aspergillus* sp. with 07 (Pe-1/Va-6), *Beauveria* sp. with 05 (Pe-5/Va-0), *Trichoderma* sp. with 05 (Pe-4/Va-1), *Fusarium* sp. with 05 (Pe-3/Va-2), *Metarhizium* sp. with 04 (Pe-3/Va-1) and *Scopulariopsis* sp. with 01 (Pe-1/Va-0) (**Figure 1**).

The species level identification of 63 isolates revealed: *Purpureocillium lilacinum* (Pe-6/Va-29); *Penicillium citrinum* (Pe-2/Va-4), *Penicillium decumbens* (Va-5), *Penicillium expansum* (Pe-2), *Penicillium pinophilum* (Pe-2), *Penicillium oxalicum* (Pe-1); *Aspergillus versicolor* (Pe-1/Va-3), *Aspergillus fumigatus* (Va-1), *Aspergillus clavatus* (Va-1), *Aspergillus sydiwii* (Va-1); *Metharhizium anisoplae* (3Pe-3/Va-1); *Scopulariopsis brevicaulis* (Pe-1). A group that did not produce any known spores was identified as *Mycellia sterilia* (4Pe, 21 Va).

### Antagonistic activity of fungal species against *Sporothrix brasiliensis*

Three species found present in both municipalities (*P. lilacinum; A. versicolor; M. anisopliae*) were chosen for the screening tests. However, it was not possible to measure the inhibition potential of isolates of *A. versicolor* and *M. anisopliae* because their colonies overgrowth time was less than 7 days. On the other hand, ten isolates of *P. lilacinum* were tested and inhibited the radial mycelial growth of *S. brasiliensis*. The mean percentage inhibition of radial growth values (PIRG) ranged from 8% to 25% (**Table 1**).

**Table 1:** Inhibition (%) of growth of *Sporothrix brasiliensis* by antagonistic *Purpureocillium lilacinum* on potato dextrose agar at 7 days.

Isolates	PIRG (%)
LTBBF-PL1	18.51
LTBBF-PL2	11.11
LTBBF-PL3	8.00
LTBBF-PL4	21.73
LTBBF-PL5	25.00
LTBBF-PL6	21.70
LTBBF-PL7	8.00
LTBBF-PL8	15.38
LTBBF-PL9	18.18
LTBBF-PL10	15.38

**Figure 2:**
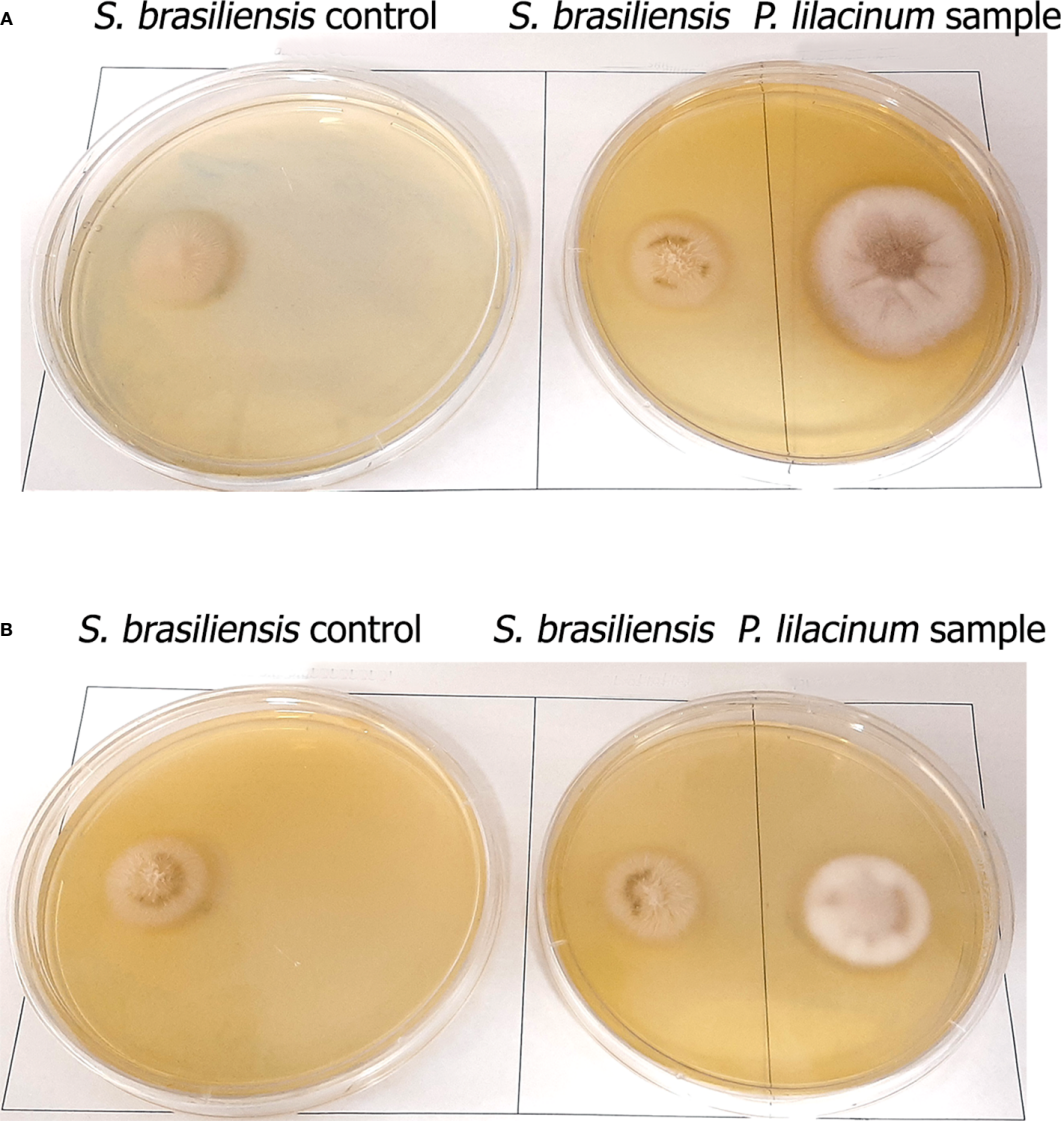
Antagonistic effects of *Purpureocillium lilacinum* isolates against *Sporothrix brasiliensis* in dual culture on the 7^th^ day of incubation. Control plates on the left showing *S. brasiliensis* colony; dual culture plates on the right showing growth inhibition of *S. brasiliensis* by *P. lilacinum*: **(A)** LTBBF-PL4 (21.73% inhibition); **(B)** LTBBF-PL9 (18.18% inhibition).

To illustrate the fungal antagonistic effect, the **Figure 2** shows the growth inhibition of *S. brasiliensis* isolate by *P. lilacinum* isolates, LTBBF-PL4 (21.73%) and LTBBF-PL9 (18.18%), on the 7^th^ day of incubation.

**Figure 3:**
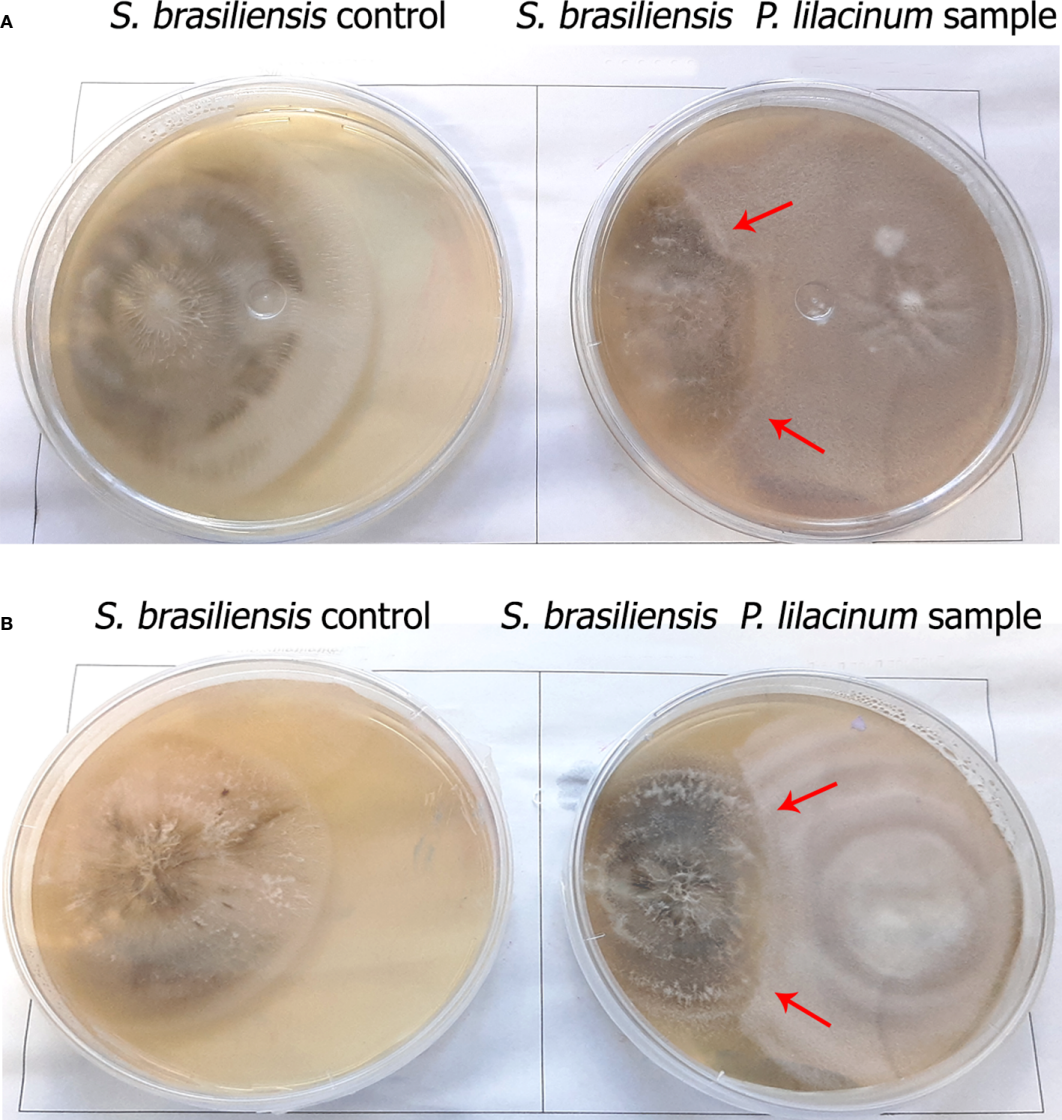
Illustration of the interaction observed between two *Purpureocillium lilacinum* isolates (**A** LTBBF-PL4; **B** LTBBF-PL9) and *Sporothrix brasiliensis* on the 28^th^ day of incubation. Control plates on the left show the growth of *S. brasiliensis* isolates and on the right plates show the interaction type “overgrowth by antagonist” which *S. brasiliensis* ceased its growth and was overgrown by *P. lilacinum* (arrows).

The interaction between the two species revealed a pattern described by Porter (1924) apud Skidmore and Dickinson (1976) as overgrowth by antagonist (**Figure 3**).

## Discussion

In the last two decades, the state of Rio de Janeiro has become a hyperendemic area of cat-transmitted sporotrichosis, mainly caused by *S. brasiliensis* (Gremião et al., 2020). Although in Brazil the species with the highest occurrence is *S. brasiliensis*, its isolation from the environment is considered rare (Rabello et al., 2022).

Over the years, many efforts have already been made to isolate *Sporothrix* spp. from environmental sources in order to understand the ecology of these species, but it has not been an easy task resulting in low isolation rates compared to the amount of screening samples (Howard and Orr, 1963; Mackinnon et al., 1969; Kenyon et al., 1984; Dixon et al., 1991; Sanchez-Aleman et al., 2004; Mendoza et al., 2007; Metha et al., 2007; Criseo and Romeo, 2010; Cruz Choappa et al., 2014; Rodrigues et al., 2014) or unsuccessful isolations (Poester et al., 2018). Recently, Rabello et al. (2022) investigated the presence of *Sporothrix* spp. in samples of abandoned demolition woods collected from a house in Petrópolis, Rio de Janeiro, Brazil, which had a petcat with sporotrichosis. All efforts resulted in only one colony identified as *S. brasiliensis* isolated directly from the cat, not the environment in which it lived. Other environmental investigation collected soil samples from rural areas of two cities, Seropédica and Nova Iguaçu, Rio de Janeiro, Brazil, where zoonotic sporotrichosis is endemic (Almeida-Silva et al., 2022). However, no fungal growth compatible with *Sporothrix* spp. was observed by authors, but molecular techniques demonstrated the presence of DNA for *S. brasiliensis* in all samples. It is important to emphasize that the initial aim of this work was the attempt to isolate *Sporothrix* spp. from the soil of an endemic region, clarifying the gap of these studies mentioned above that, the same way as observed by us, were also unable to isolate the fungus. However, we recognize that the lack of use of molecular tools constitutes a deficiency of this study. Therefore, one of the future steps of our studies is to use these methodologies in order to refine our search for fungal species of interest.

In the present study we were also unsuccessful in isolating *Sporothrix* spp. from the soil, and the question is: what makes this isolation difficult? What kind of interaction in the soil could be hindering the growth of some species? Therefore, in an attempt to understand what would hinder the growth of *Sporothrix* spp., we decided to study *in vitro* the interaction of some *P. lilacinum* isolates (the species most isolated from the soil evaluated here and whose results were quantified) with one *S. brasiliensis* isolate. To understand how fungi, especially in the soil, interact, it is necessary to be aware that fungi grow in competition with other microorganisms and can overcome competition by rapid growth, sporulation, stress recovery, and the use and negation of inhibitors (Knowles et al., 2022). According to Almeida-Silva et al. (2022) some factors, among them, fungal faster growth rates, can limit the growth of pathogenic fungi, for example, *Sporothrix* spp. One of the strategies used by fungi in competition for habitat is the so-called antagonist effect. This effect is detected during mycelial interaction but can occur without mycelial contact, by the production of volatile and diffusible organic compounds (Boddy and Hiscox, 2016). In our experiment, was verified and quantified the inhibition *in vitro* of one *S. brasiliensis* isolate by ten *P. lilacinum* isolates showing different percentage of inhibition. Variation in ability to inhibit fungal isolates was also observed by Sonkar (2018) who described excellent antagonistic potentials differing between *Trichoderma* strains against *Fusarium oxysporum*. Isolates of *Trichoderma* antagonized *Ceratocystis paradoxa* growth and different strains also showed different degrees of inhibition (Rahman et al., 2009).

Previous studies point to the use of *P. lilacinum* as a biocontrol agent, since it is described as insect parasites (Barra et al., 2015; Hotaka et al., 2015) and nematodes (Singh et al., 2013; Liu et al., 2014). In addition, researchers have reported the ability of this species to inhibit the growth of other fungi, such as *Penicillium digitatum* (Elsherbiny et al., 2021) and *Verticillium dahliae* (Lan et al., 2017). But, how does this species interact, *in vitro*, with one of the agents of sporotrichosis? Our experiments demonstrated that the colony of *S. brasiliensis* ceased its growth and was overgrown by *P. lilacinum*, interaction classified as “overgrown antagonist” (Skidmore and Dickinson, 1976). Liu et al. (2020) noticed that *P. lilacinum* inhibited the growth of *Botritys cinerea* without overlapping or even physical contact between the two fungi, but they evaluated this interaction only by 7 days. On the 7^th^ day, in our experiment, it was also observed that all *P. lilacinum* isolates did not overlap the *S. brasiliensis* colony (**Figure 2**), only doing so during the 28 days of incubation.

One of the possible mechanisms for antagonism between fungi is the production of organic compounds, so-called secondary metabolites that can alter spore germination, mycelial morphology, foraging behavior, and enzyme production (Boddy and Hiscox, 2016). Therefore, it is plausible to suppose that compounds produced by the *P. lilacinum* isolates tested here may have inhibited the growth of *S. brasiliensis* isolate. This hypothesis is supported by the study of Liu et al. (2020) who discovery a new antifungal lipopeptaibol (leucinostatin Z) from *P. lilacinum* against *B. cinerea* after co-culturing of two fungi on agar plate. In addition, *P. lilacinum* is reported as a species that produces different metabolites already characterized as leucinostatins, paecilotoxin and mycotoxin (Mikami et al., 1989; Khan et al., 2003; Park et al., 2004; Sharma and Sharma, 2016).

In summary, our results confirm the difficulty in isolating *Sporothrix* spp. from environmental sources and point out that very likely fungal species with very fast growth and capable of producing metabolites are the agents of this difficulty. Furthermore, this study paves the way for the isolation, purification and future identification of *P. lilacinum* secondary metabolites with biological activity that could be tested against pathogenic fungi.

## Data availability statement

The original contributions presented in the study are included in the article/supplementary material. Further inquiries can be directed to the corresponding authors.

## Author contributions

GC, AA and IF carried out experiments. AM wrote the original manuscript. DC-M analyzed the data. DC-M and CB wrote the manuscript. MO designed the study, acquired funding and administrated the project. DC-M, SP, CB and MO revised the manuscript. All authors contributed to the article and approved the submitted version.

## Funding

This work was supported by Fundação Carlos Chagas Filho de Amparo à Pesquisa do Estado do Rio de Janeiro (FAPERJ - Grants: JCNE E-26/203.301/2017, JCNE E-26/201.433/2021– MO; E-26/202.737/2019 - SP), CAPES (DC-M fellowship 88882.317297/2019-01), and Conselho Nacional de Desenvolvimento Científico e Tecnológico (CNPq - Grant Proc. 409227/2016-1 – MO; CNPq 312238/2020-7 - SP).

## Acknowledgments

We are grateful to Fiocruz and all state funding agencies to support this project.

## Conflict of interest

The authors declare that the research was conducted in the absence of any commercial or financial relationships that could be construed as a potential conflict of interest.

## Publisher’s note

All claims expressed in this article are solely those of the authors and do not necessarily represent those of their affiliated organizations, or those of the publisher, the editors and the reviewers. Any product that may be evaluated in this article, or claim that may be made by its manufacturer, is not guaranteed or endorsed by the publisher.
